# Evaluation of One- and Two-Step Impression Techniques and Vertical Marginal Misfit in Fixed Prothesis

**DOI:** 10.1155/2023/9898446

**Published:** 2023-02-21

**Authors:** Sina Radfar, Marzieh Alikhasi, Sotude Khorshidi, Saba Tohidkhah, Reza Morvaridi Farimani, Sima Shahabi

**Affiliations:** ^1^School of Dentistry, Tehran University of Medical Sciences, Tehran, Iran; ^2^Dental Research Center, Dentistry Research Institute, Tehran University of Medical Sciences, Tehran, Iran; ^3^Department of Prosthodontics, School of Dentistry, Tehran University of Medical Sciences, Tehran, Iran; ^4^Department of prosthodontics, Dental branch, Islamic Azad University, Tehran, Iran; ^5^Minnesota Dental Research Center for Biomaterials and Biomechanics, School of Dentistry, University of Minnesota, Minneapolis, USA; ^6^Department of Dental Biomaterials, School of Dentistry, Tehran University of Medical Sciences, Tehran, Iran

## Abstract

**Methods:**

12 impressions were made of a resin maxillary model (second premolar and second molar) with two prepared abutment teeth using vinyl polysiloxane (VPS); the margin of the second premolar was 0.5 mm subgingivally; and the margin of the second molar tooth was at the level of the gingiva. Impressions were made using two techniques: one-step and two-step putty/light materials. A three-unit metal framework was fabricated on the master model using the computer-aided design/computer-aided manufacturing (CAD/CAM) technique. The vertical marginal misfit was evaluated in the buccal, lingual, and mesial and distal surfaces of the abutments on the gypsum casts using a light microscope. Data were analyzed using the independent *t*-test (*α* < 0.05).

**Results:**

The results showed significantly lower vertical marginal misfit in all six areas evaluated around the two abutments in the two-step impression technique compared with the corresponding values in the one-step technique.

**Conclusion:**

Vertical marginal misfit in the two-step technique with a preliminary putty impression was significantly lower than in the one-step putty/light-body technique.

## 1. Introduction

The clinical durability of restorations depends on their precise adaptation to the underlying tooth structure [1, 2]. An accurate impression is required to fabricate a precise restoration. In cases of discrepancy and misfit, cement dissolution occurs, which leads to the development of caries, periodontal disease, and the failure of restorations [3]. A number of factors could affect the accuracy of impressions, such as the impression technique, type of impression material, thickness of the impression material, and correct application of materials [4, 5]. A precise impression material with optimal dimensional stability is imperative to record the details of the prepared tooth [6]. VPS is a commonly used impression material in fixed prosthodontics, which has low polymerization shrinkage and high-dimensional stability [7]. Now, with the development of impression materials, it appears that the impression technique has a greater impact on dimensional accuracy and the accurate recording of details than impression material. Controversy exists regarding the greater effect of the impression material or impression technique on the accuracy of impressions [3]. Silicon materials have different consistencies for use in different impression techniques. Several impression techniques are available, which differ in terms of the type of impression material and spacers used [8]. In the two-step technique, first, a putty impression is made to provide space for the light body, and then, the final impression is made using the light body. Several methods can be employed to create space in the two-step technique [9]. One suggested strategy for this purpose is to make a putty impression, relieve (cut out) the putty material at the finish line, and make a final impression with the light body [9]. In the one-step technique, the base and catalyst of the putty material are mixed in ratios recommended by the manufacturer. Then, the light body is injected around the prepared tooth by a syringe, and the putty is applied in a tray [10]. There are several problems with both of these two-step impression techniques. The first issue is the fact that the thickness of the light body cannot be practically controlled, and we may observe that in some marginal impression areas, the putty has pushed aside the light body and the margin is recorded by the putty. The second reason is that the composition of putty contains materials of high elasticity, which can be exposed to hydraulic pressure and change; this change would not be apparent until the casting made from the mold has resided [10].

Several methods are available to assess marginal discrepancy, such as determining reference points on the original model and measuring the distance between these points [11]. Another technique is to fabricate a metal die on the abutment of the original model and its subsequent placement on the plaster cast and assessment of adaptation of metal die margin with the finish line on the plaster cast under a light microscope [12]. Clinical examination by a dental mirror and an explorer and the use of a silicon replica are among other methods available for this purpose [13].

Considering all the above and a search of the literature, many studies have assessed and compared the accuracy of the two-step impression technique with a spacer and the one-step impression technique [12, 14–16]. However, only a few studies have compared one-step versus two-step impression techniques in terms of accuracy. This study aimed to compare the one-step and two-step impression techniques and the effect of preparation of putty impression on the marginal discrepancy of a three-unit metal framework.

## 2. Materials and Methods

**Figure 1:**
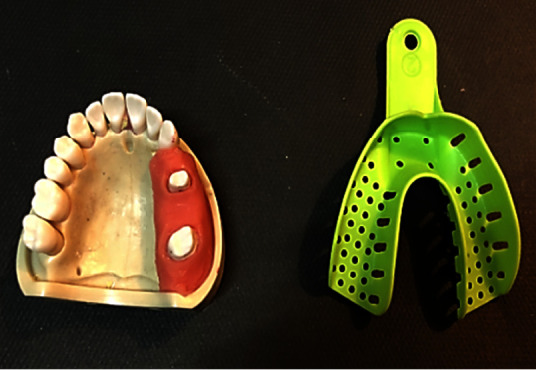
The original model with the resin as soft tissue. A plastic tray was used to prepare the impression.

A maxillary model of the maxilla (Prosthetic Restoration Jaw Model, Nissin, Kyoto, Japan) with two abutments (second premolar and second molar) and a pontic space at the site of the first molar was used in this study. The soft tissue of the resin model was designed by the additional silicon specifically designed for this purpose (Gingival Mask, Feguramed GmbH, Buchen, Germany) (Figure 1). The finish line of the second premolar was located 0.5 mm subgingivally, and the second molar tooth was at the level of the gingiva. The prepared teeth had a chamfer finishing line.

The original model was scanned by an intraoral scanner, and a three-unit metal framework was designed using the CAD/CAM system and milled using a cobalt-chromium block. Next, the metal framework was seated on the original model, and its clinical adaptation was evaluated by a technician. A reference point with some distance from the margin was marked in the mesial, distal, lingual, and buccal surfaces of the abutment teeth not to damage the margin.

**Figure 2:**
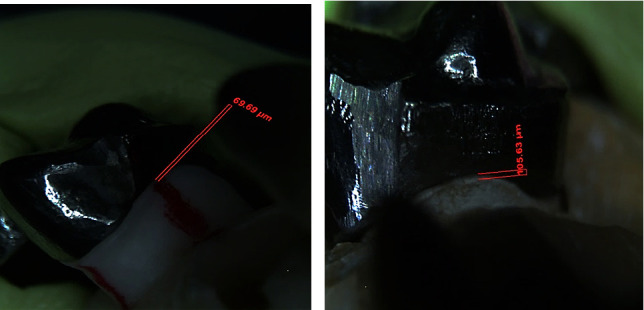
The vertical marginal misfit was evaluated using a light microscope at ×10 magnification under a direct LED lamp in the midbuccal, midmesial, and midlingual around the second premolar abutment. (a) The vertical misfit calculation in the mesial surface of second premolar on the original model. (b) The vertical misfit calculation in the mesial surface of second premolar on the cast model for the two-step putty/light-body technique.

The soft tissue designed to simulate the gingiva was removed from the original model. Next, the metal framework was placed on the abutments in the original model with gentle finger pressure and fixed with the putty material. The vertical marginal misfit was then evaluated using a light microscope (SZX16, Olympus, Japan) at ×10 magnification under a direct LED lamp in the midbuccal, midmesial, and midlingual around the second premolar abutment as a standard (Figure 2). Considering the presence of a pontic space, it was not possible to measure the vertical marginal misfit at the distal surface of the second premolar and the mesial surface of the second molar. The vertical marginal misfit at the designated points was analyzed using software (Carl Zeiss AxioVision Microscopic Imaging Software Release 4.8, Germany).

According to a previous study [12], the minimum sample size was calculated to be 9 in each study group, using the two-sample*t*-test power analysis (SPSS 19), assuming alpha = 0.5, beta = 0.2, a mean difference of 13, and standard deviations of 10.25 and 7.06. In order to increase the reliability of our study, twenty-four quadrant impressions were made with additional silicon using the one-step putty/light body (#12) and two-step putty/light-body (#12) techniques. Prefabricated perforated plastic trays were used for impression making.

In the one-step technique, the putty and light body (Duo sil, Bukwang, Busan, Korea) were used simultaneously. Both putty and light body were mixed simultaneously according to the manufacturer's instructions. The putty material was applied into a tray, and the light body was injected directly around the abutments using an automixing gun dispenser. The tray was placed on the cast and kept in place with hand pressure for 10 minutes. The impression was then removed from the cast. In the two-step putty/light-body technique, a putty impression was made. For this purpose, the putty material was prepared according to the manufacturer's instructions and applied to the tray. The tray was placed on the original model with hand pressure and compressed for 10 minutes until the material set and was removed from the cast. The putty was cut out by 2 mm at the marginal area of the second premolar, and the light body was then injected around the abutment teeth. The putty impression was placed again on the model, and a 12-minute time was allowed to set.

All impressions were kept at 25°C temperature for one hour prior to pouring. They were then poured with type IV dental stone (Welmix G30, Asia Chemi Teb, Tehran, Iran). To prepare the dental stone, 50 g of gypsum was mixed with 10 mL of water according to the manufacturer's instructions. It was first mixed manually and then placed on the auto-mix vacuum (Auto mix II, KFP-Dental, Tehran, Iran) in order to eliminate voids. The impressions were poured, one-hour time was allowed for the primary setting, and the casts were separated from the impressions. After 24 hours, the casts were evaluated under a light microscope to assess the vertical marginal misfit.

Vertical marginal misfit was evaluated for each of the 24 plaster casts three times. The amount of vertical marginal misfit in each of the measures was subtracted from the vertical marginal misfit of the original model in the two impression techniques. The mean and standard deviation values were calculated and analyzed using the independent *t*-test.

## 3. Results

**Figure 3:**
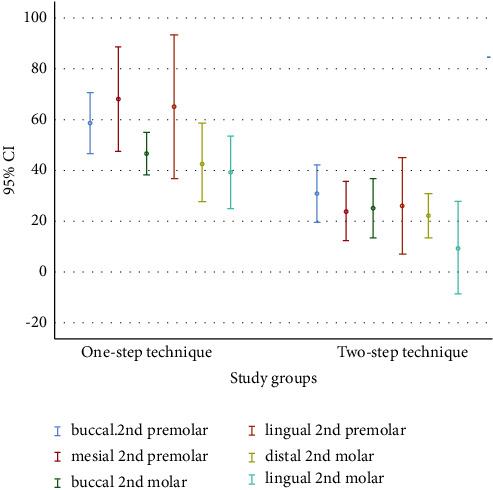
The average error chart and 95% on confidence internal of one-step and two-step methods.

The following results were obtained by measuring the vertical marginal misfit in the two groups on plaster casts: The vertical marginal misfit in the two-step technique was 93.57 ± 18.42 *μ*m, 96.00 ± 17.59 *μ*m, and 102.39 ± 29.92 *μ*m at the midmesial, midbuccal, and midlingual of the second premolar and 98.96 ± 13.23 *μ*m, 101.57 ± 18.26 *μ*m, and 82.43 ± 28.52 *μ*m in the middistal, midbuccal, and midlingual of the second molar, respectively. The vertical marginal misfit in the one-step technique was 137.81 ± 32.37 *μ*m, 123.84 ± 19.14 *μ*m, and 141.46 ± 44.57 *μ*m at the midmesial, midbuccal, and midlingual of the second premolar and 123.03 ± 13.27 *μ*m, 119.94 ± 24.76 *μ*m, and 112.11 ± 22.5 *μ*m in the middistal, midbuccal, and midlingual of the second molar, respectively (Table 1 and Figure 3). The independent *t*-test showed that the vertical marginal misfit in all areas in the two-step technique was significantly smaller than in the one-step technique (*P* < 0.05).

## 4. Discussion

This study assessed the accuracy of two putty/wash impression techniques by measuring the vertical marginal misfit under a light microscope. The results showed that the two-step putty/light-body impression technique yielded a smaller gap between the margin of the three-unit framework and the finish line. The mean amount of vertical marginal misfit in this technique was 95 *μ*m, closer to the vertical marginal misfit in the original model, 72 *μ*m. This value was 125 *μ*m in the one-step technique.

Basapogu et al. [16] reported different results in their study. They demonstrated that the accuracy of the one-step putty/wash impression technique was similar to that of the two-step putty/wash technique with a polyethylene spacer. Since the impression material used in both studies was the same, the difference observed in the results could be due to the methodology. They used a polyethylene spacer to create space for the wash in the two-step technique, whereas we cut out the putty impression with a scalpel in order to create space for the light body. Moreover, they marked some reference points on the two abutments and evaluated the distance between them and between points on the same abutment, as well as the height and diameter of the abutments. However, we fabricated a three-unit metal framework with the CAD/CAM technology and assessed its marginal discrepancy under a light microscope. The model used in the two studies was also different.

Regarding the effect that the type of spacer might have on impression accuracy, Mann et al. [17] conducted a study assessing the accuracy of the two-step putty/wash impression technique with an aluminum wrap spacer and the two-step technique with the traditional cut out of putty impression. Although the impression material and the model used in both these studies were similar, they reported that using the aluminum wrap as a space maintainer in the two-step technique yielded higher dimensional accuracy in gypsum dies than with no use of aluminum wrap and traditional cut out. Another difference between the two studies, which may cause the difference in results, is the method of measuring the marginal misfit. In the present study, a light microscope was used to measure the marginal discrepancy while using a 3D coordinate-measuring machine for their measurements. In another study, a plastic wrap spacer was used with the two-step putty/wash impression technique, and the results were in agreement with ours. The two-step putty/wash impression technique, with a plastic wrap spacer, was more accurate than the one-step putty/wash technique [15].

On the other hand, Nissan et al. [12] compared the marginal discrepancies between the two-step putty/wash and one-step putty/wash techniques. Impression material, impression techniques, and measurement method were the same as in our research. According to their report, the two-step technique and the creation of a 2 mm space yielded a lower gap compared with the one-step impression technique, which is in line with our results. In addition to the studies mentioned above, there are still a couple of other reports [14, 18] that tried to assess the accuracy of the two-step putty/wash and the one-step putty/wash techniques concerning the vertical discrepancy. Disregarding the minor methodological differences, most of them presented the same results as we did in the current study, that the two-step technique was more accurate than the one-step technique.

This study is an in vitro design. Thus, it was not possible to assess the effects of factors such as blood, saliva, oral temperature, and clinical setting environment on the accuracy of impression techniques. Considering our methodology, this study only allowed measurement of a marginal discrepancy, and assessment of internal fit and changes in height and diameter of the dies or the distance between them (which are all important) was not feasible. However, a number of confounders such as fabrication of the resin pattern, flasking, and type of metal can affect the accuracy of the final restoration. Therefore, further clinical studies are required to elucidate the role of the impression technique and factors affecting the accuracy of impressions.

## 5. Conclusion

Measurement of vertical marginal misfit under a light microscope revealed that this value was 95 *μ*m in the two-step putty/light-body technique; this value was clinically acceptable (50–120 *μ*m) [12]. The vertical marginal misfit in the one-step putty/light-body impression technique with the addition of silicon was 125 *μ*m, which can contribute to a marginal misfit following cementation and other steps that may cause distortion (casting, sintering of porcelain, and polishing). Vertical marginal misfit in the two-step technique with a preliminary putty impression was significantly lower than that in the one-step putty/light-body technique.

## Figures and Tables

**Table 1:** The independent *t*-test results of the vertical marginal misfit comparing each surface between one-step and two-step techniques.

Group	Mean (*μ*m)	Std. deviation	*P* value
Mesial of 2^nd^ premolar, one-step technique	137.8125	32.3716	0.001
Mesial of 2^nd^ premolar, two-step technique	93.57	18.427	
Buccal of 2^nd^ premolar, one-step technique	123.845	19.141	0.001
Buccal of 2^nd^ premolar, two-step technique	96.005	17.597	
Lingual of 2^nd^ premolar, one-step technique	141.468	44.571	0.019
Lingual of 2^nd^ premolar, two-step technique	102.394	29.929	
Buccal of 2^nd^ molar, one-step technique	123.035	13.274	0.003
Buccal of 2^nd^ molar, two-step technique	101.57	18.268	
Distal of 2^nd^ molar, one-step technique	119.945	24.768	0.0167
Distal of 2^nd^ molar, two-step technique	98.967	13.233	
Lingual of 2^nd^ molar, one-step technique	112.16	22.505	0.009
Lingual of 2^nd^ molar, two-step technique	82.436	28.526	

## Data Availability

The data used to support the findings of this study are available from the corresponding author upon request.
